# Stress, burnout and work engagement among physicians of the state of Paraná, Brazil

**DOI:** 10.47626/1679-4435-2022-842

**Published:** 2023-08-08

**Authors:** Maria Bernadete Gonçalves, Ana Maria Benevides Pereira, Pedro Guilherme Basso Machado

**Affiliations:** 1 Departamento de Medicina, Universidade Estadual de Maringá, Maringá, PR, Brazil; 2 Departamento de Psicologia, Universidade Estadual de Maringá, Maringá, PR, Brazil; 3 Centro Universitário Autônomo do Brasil, Curitiba, PR, Brazil

**Keywords:** burnout syndrome, occupational stress, work engagement, síndrome de *burnout*, estresse ocupacional, engajamento no trabalho

## Abstract

**Introduction:**

Precariousness of medical work, with loss of autonomy and devaluation, in addition to unstable and non-guaranteed employment bonds, has caused health problems in professionals, hampering the assistance provided. Research shows a high prevalence of stress, burnout, depression, and suicide among physicians. This study investigated aspects of mental health in physicians from the state of Paraná, Brazil.

**Objectives:**

We aimed to evaluate indicators of stress, burnout, and work engagement measured by inventories specifically designed for each one: Stress Symptoms Inventory, Burnout Syndrome Inventory, Utrecht Work Engagement Scale, respectively.

**Methods:**

The professionals answered the questionnaires remotely, after accepting the invitation for the study and signing the consent form.

**Results:**

A total of 1,201 physicians answered the questionnaires, with mean age of 37 years; 53.9% of participants were women; 63.5% graduated in Paraná. Of the total number of participants, 97.5% and 93.4% presented psychological and physical symptoms of stress, respectively. According to the Inventário da Síndrome de Burnout, the prevalence of diagnosis of burnout was estimated at 59.4%. As for work engagement, 40% of participants showed very high levels in the overall score of the construct.

**Conclusions:**

Most physicians showed signs of stress; burnout rates were high; negative organizational conditions prevailed in the work environment; work engagement was frequent.

## Abstract (pt)

**Introdução:**

A precarização do trabalho médico, com a perda de autonomia e a desvalorização, além de vínculos empregatícios instáveis e sem garantias, tem provocado problemas de saúde nos profissionais, prejudicando a assistência prestada. Pesquisas mostram elevada prevalência de estresse, *burnout,* depressão e suicídio entre médicos. Este estudo investigou aspectos da saúde mental de médicos do estado do Paraná, Brasil.

**Objetivos:**

Pretendeu-se avaliar indicadores de estresse, *burnout* e engajamento no trabalho, aferidos pelos inventários elaborados especificamente para cada um: Inventário de Sintomatologia de Estresse, Inventário da Síndrome de Burnout, Utrecht Work Engagement Scale, respectivamente.

**Métodos:**

Os profissionais responderam remotamente aos questionários, após aceitar o convite e assinar o termo de consentimento.

**Resultados:**

Responderam 1.201 médicos, com média de idade de 37 anos; 53,9% dos participantes eram mulheres; 63,5% se graduaram no Paraná. Do total de participantes, 97,5% e 93,4% apresentaram sintomas psicológicos e físicos de estresse, respectivamente. De acordo com o Inventário da Síndrome de Burnout, a prevalência do diagnóstico de *burnout* foi estimada em 59,4%. Quanto ao engajamento, 40% apresentaram nível muito alto na escala geral do constructo.

**Conclusões:**

A maioria dos médicos manifestou sinais de estresse; foi elevada a taxa de *burnout;* predominaram condições organizacionais negativas no ambiente de trabalho; o engajamento no trabalho foi frequente.

## INTRODUCTION

Valued for its social role, medicine has been discredited and undergone radical changes in its practice. Precarious working conditions leading to suffering, stress, and anxiety are a no longer questioned reality in medical professional practice, since work organization is the main driving force of workers’ mental life.^1,2^

Medical work is characterized by excessive workload, extended working shifts, long-distance duties, and little autonomy, and physicians have an average of two to four employment bonds, reaching up to 20 bonds. This situation generates several ethical and technical problems, in addition to increased levels of burnout, illness, and frequency of involvement in traffic accidents with victims, sometimes fatal.^1,2^

Numerous problems generate stress in physicians, including idealized behavior, intense and frequent contact with pain, suffering, death, and dying; dealing with intimacy and with difficult patients; uncertain and limited medical knowledge.^1,2^ Nonetheless, physicians continue working with dedication, and the number of individuals dropping out the medical profession or the medical school is low, possibly because this profession maintains an “artistic” component,^1^ and medicine has been historically considered a ministry.

Stress, conceptualized as a “general adaptation syndrome,” is related to a strategy of the human body to adapt to changes, demands, disappointments, and other everyday events. People usually experience a certain level of stress, only varying in its intensity.^3,4^ Inability to overcome stressful experiences wears individuals down, and the results of this process depends on its degree, frequency, and duration.^4^

Stress is manifested in stages: in the beginning, individuals encounter a source of stress, then attempt to recover themselves from physiological changes, returning to balance; failure to achieve this balance leads to exhaustion. It is a dynamic process in which thoughts, feelings, behaviors, and biophysiological mechanisms attempt to adapt themselves.^3,4^ The results of the response will depend on individual and social differences, cultural characteristics, and individual behavioral adaptive patterns.^4^

Stress at the workplace is unavoidable, due to the natural contemporary demands. According to the World Health

Organization (WHO),^5^ occupational stress is manifested when professionals are presented with demands and pressures that are not matched to their knowledge and abilities and which challenge their ability to cope. These demands may occur at the organizational level, such as company’s culture and structure; at the group level, such as scarce teamwork and rivalries; and at the individual level, such as unclear and conflicting roles, limited work.

Physicians and nurses are more prone to chronic occupational stress, because they are exposed to multiple factors and sources of stress in their everyday work.^3,6^ Individuals adaptation to a stressful situation requires coping strategies, not always leading to good results.^3^ When these strategies fail, diseases such as burnout syndrome may occur.^7,8^

Organizational factors are extremely important in the study of burnout syndrome, because they may provide resources for the maintenance/promotion of well-being at work, but they may have an opposite impact if these resources are lacking. Organizational conditions may be positive (POC), which are factors that facilitate activities and associated with organizational resources and work engagement; or negative (NOC), associated with occupational costs and demands and related to burnout and stress.^7^

Burnout syndrome is characterized by high levels in three dimensions: emotional exhaustion (EE), a manifestation of chronic stress; depersonalization or dehumanization (DEs), formerly known as “cynicism”, characterized by negative attitudes; and reduced professional accomplishment (rPA), defined as reduced productivity, low morale, and inability to work.^8,9^ This syndrome is manifested as organic or psychological symptoms, such as sleep disturbances, muscular or musculoskeletal pain, headache, gastrointestinal or cardiovascular disorders, among others, in addition to symptoms involving feelings and emotions.^1,9^

Among physicians, burnout syndrome affects quality of care, safety, disease evolution, and degree of patients’ satisfaction; at work, it interferes with team dynamics and with institutions financial health.^10^

Bianchi et al.^11^ believe that EE is more associated with depressive symptoms and that chronic stress at work may lead to burnout and cause depression; they added that antidepressants improve burnout.^11,12^ Schonfeld & Bianchi^12^ add that they found an association between burnout and depressive symptoms and anxiety According to Maslach & Leiter,^9^ burnout syndrome is a specific occupational dysphoria and depression is a mental disease different from burnout. Oquendo et al.^13^ believe that there is a reluctance to diagnose depression in physicians; therefore, they refer to burnout, a problem related to the workplace that is not an endogenous condition requiring psychiatric treatment.^13^

Despite extensive evidence that burnout, depression, suicide, and abuse of licit and illicit substances are more prevalent among physicians than in the general population, the mental health of these professionals has not received the required attention.^6,13,14,15^

With the emergence of positive psychology, which privileges the study of health aspects, work engagement emerges as a construct referring to a positive cognitive state, present over time, of a motivational and social nature.^16,17^ It consists of three dimensions: Vigor (VI) – behavioral and energetic factor, manifested by high levels of energy and resilience; Dedication (DE) – an emotional factor related to a sense of significance and challenge; and Absorption (AB) – high concentration and absorption in activities.^16,18^

Engaged professionals are more likely to be committed to activities and have satisfactory results both quantitatively and qualitatively, in addition to being less likely to develop burnout syndrome.^17^^19^

This research aimed to evaluate the prevalence of symptoms of stress and burnout syndrome, as well as organizational conditions and work engagement, among physicians working in the state of Paraná, Brazil.

## METHODS

This is an empirical, cross-sectional, quantitative, *ex post facto,* descriptive study.

In October 2018, an invitation letter was sent to all physicians registered at Paraná’s Regional Council of Medicine (Conselho Regional de Medicina do Paraná, CRM-PR) up to February 1^st^, 2017. A link was made available to those who agreed to participate in the research, in order for them to access the research protocol and answer it online. Due to the low adherence, a new letter was sent 30 days later, emphasizing the importance of the study, with a 10-day deadline to answer the questionnaires.

The protocol, made available through the Qualtrics platform, contained a social and professional questionnaire followed by questions related to the specific tests: Stress Symptoms Inventory (Inventário de Sintomatologia de Estresse, ISE),^20^ Burnout Syndrome Inventory (Inventário da Síndrome de Burnout, ISB),^7^ and Utrecht Work Engagement Scale (UWES).^16^

The ISE comprises 27 statements considered to indicate stress, subdivided into Physical Symptoms (PhyS, 7 items), e.g., “I have been feeling fatigue,” and Psychological Symptoms (PsyS, 20 items), e.g.,: “I feel angry and impatient,” measured by a 5-point Likert scale, with 0 corresponding to “never” and 4 corresponding to “frequently”.

The ISB, developed in Brazil to cover several areas of occupational practice, has two parts: the first one assesses individuals’ perception of their work environment (“antecedent factors”), with POC items (related to a good work environment), e.g.: “I feel that I am effectively part of a working team,” and NOC (defining an unfavorable work environment), e.g.: “Bureaucracy takes most of my time at work.” The second part evaluates burnout syndrome and includes 19 items distributed into the following dimensions: EE (n = 5), e.g.: “I feel that my work has consumed all my energy”; PA (n = 5): “My work fulfills me professionally”; DEs (n = 4): “I’ve had to toughen up to maintain my job;” and emotional withdrawal (EW) (n = 5): “I realize that I avoid closer contact with people at work.” To diagnose the syndrome, individuals should have high scores in EE, DEs and EW dimensions, and low scores in the PA dimension.^3,7^

The UWES, version for workers,^16^ assesses work engagement in general and in specific dimensions: VI (n = 6), such as “At my work, I feel bursting with energy”; DE (n = 5), “I find the work that I do full of meaning and purpose“; and AB (n = 6), “Time flies when I’m working.” Answers were given on 7-point Likert scale ranging from 0 to 6 (0 = never and 6 = always/everyday). The instrument and its manual were translated and adapted by Agnst et al.^21^

Participation in the research was voluntary, and only active medical professionals who answered the questions related to social and professional identification and to the instruments were considered. The participants were informed that they would not be identified and that information would be processed as one data set.

### STATISTICAL ANALYSIS

Results were calculated using the SPSS, version 21, and AMOS, version 18 statistical software. Descriptive analysis [means (M), standard deviation (SD), and calculation of scores for each instrument], reliability (Cronbachs alpha), correlation analysis (Pearson correlation), difference in means (Student’s t test and ANOVA), and confirmatory factor analysis (structural equation modeling). A 95% confidence interval was used in all analyses.

### ETHICAL CONSIDERATIONS

The work complied with the standards of Resolution no. 466/12 of the Brazilian National Health Council and was approved by the Research Ethics Committee of a higher education institution (HEl) under number CAE 2269960, in September 2017, on Plataforma Brasil.

## RESULTS

Among the 23,524 physicians to whom the questionnaire was sent, only 1,334 (5.7%) returned it, some of them partially completed, and 133 physicians were excluded because they only answered the identification questions. The 1,201 (5.1%) participants who answered most questions were included in the analyses, in order to avoid further losses; thus, the total (n) is not the same for the different aspects researched.

Out of the 1,201 participants, 647 (53.9%) self-reported as female, and 553 as male (46.0%); only one self-reported as belonging to another gender. Among those who informed their age, mean age was 37 years (ranging from 24 to 81 years); 770 respondents (64.2%) were 42 years or younger, whereas 104 (8.7%) were 60 years or older; 214 physicians (17.8%) reported being younger than 30 years. At the time of data collection, 880 (83.2%) out of 1,058 physicians were in a stable relationship. Among those who informed where they earned their medical degree, 762 (63.5%) studied at a HEI in the state of Paraná, 100 (8.32%) in the state of Santa Catarina, 70 (5.8%) in the state of Rio Grande do Sul, 62 (5.2%) in the state of São Paulo, and 60 (5.0%) in the state of Rio de Janeiro. The other participants reported having graduated in other Brazilian states or in other countries, such as Argentina, Bolivia, Ecuador,

Portugal, and Cuba, totaling 128 physicians (10.7% of the sample). Considering those who earned their medical degree in the state of Paraná, most of them graduated at Universidade Federal do Paraná (40.7%).

With regard to academic degree, 190 obtained an undergraduate degree; 460 completed medical residency, and 343 had a specialization (without specifying whether it was residence or another type of specialization – some participants had more than one); furthermore, 141 had a master’s degree; 67 had a doctoral degree, and 16 had a post-doctoral degree.

Furthermore, 886 respondents answered the questions related to leisure activities, and some of them reported more than one option, the most cited of which were: travelling (212; 23.9%), practicing physical activity (177; 20.0%), reading (175; 19.8%), sleeping (116; 13.1%), staying with the family (79; 8.9%), resting (59; 6.7%), seeing friends (56; 6.3%), among others.

Among the 1,058 physicians who answered most questions, 47.6% were or had already been on psycotherapeutic or psychiatric treatment; 53% reported taking medications of continuous use, and 29% used controlled medications; 74.5% believed that medical practice was a source of stress, and 46.8% have already thought in changing profession.

In the results obtained with instruments to investigate signs of stress, burnout syndrome, and engagement, there is variation in the sample population (n), because some participants did not complete the tests. Among the 1,057 who answered most items in this phase, 473 (44.7%) were men, and 584 (55.2%) were women.

As for ISE, 97.5% of the physicians had high levels of PsyS, and 93.4% of PhyS.

According to the results presented in Table 1, reliability indexes were higher than 0.7, a result considered adequate with regard to the criterion α > 0.7.

**Table 1:** Mean, SD, Cronbach’s alpha, and frequency distribution of ISE scores

	Mean	SD	α	Total (n)	Low (%)	Average (%)	High (%)
PsyS	3.0	0.2	0.9	1,058	0.0	2.5	97.5
PhyS	29	0.2	0.7	988	0.0	6.6	93.4

ISE = Inventário de Sintomatologia de Estresse; PhyS = physical symptoms; PsyS = psychological symptoms; SD = standard deviation; α = Cronbach’s alpha.

With regard to confirmatory factor analyses, results were also adequate, attesting the quality of the model for the present sample: comparative fit index (CFI) = 0.8; adjusted goodness-of-fit index (AGFI) = 0.8; root mean square error of approximation (RMSEA) = 0.1 (0.08 more specifically). Regression indexes ranged from 0.4 (ISE17ßPsyS) to 0.8 (ISB10ßPhyS), being thus higher than 0.4, which indicates that these items are constitutive components of their scales.

Results for the ISB, presented in Table 2, show high score for NOC in 91.9% of participants (n = 972); whereas scores for POC had an almost equal distribution between high (34.6%, n = 366), medium, and low scores. Mean values were lower for POC (M = 2.04; SD = 0.8) and higher for POC (M = 2.9; SD = 0.3) compared to those found in a validate study of the scale with 604 participants with different occupations.^7^

**Table 2:** Mean, SD, Cronbach’s alpha, and frequency distribution of ISB scores

Scale	Mean	SD	α	Total(n)	High(%)	Average (%)	Low(%)
POC	2.0	0.8	0.9	1,058	34.6	34.7	30.7
NOC	2.9	0.3	0.9	1,048	91.9	7.0	0.2
EE	2.9	0.3	0.9	1,058	87.1	12.9	0.0
Des	3.0	0.2	0.9	1,056	96.3	3.7	0.0
EW	2.7	0.6	0.9	1,058	76.3	17.7	6.0
rPA	1.1	0.4	0.9	1,058	2.4	9.5	88.1

Des = deshumanization; EE = emotional exhaustion; EW = emotional withdrawal; ISB = Inventário da Síndrome de Burnout; NOC = negative organizational conditions; POC = positive organizational conditions; rPA = reduced professional accomplishment; SD = standard deviation; a = Cronbach’s alpha.

With regard to burnout syndrome, most participants presented high scores in the following dimensions: EE (87.1%), DEs (96.3%), EW (76.3%) and rPA (88.1%) (Table 2).

The results for Cronbachs alpha tests were considered adequate (>0.7), all ranging around 0.9. Confirmatory factor analyses also showed adequate results, attesting the quality of the model for the present sample: CFI = 1.0/AGFI= 0.9/RMSEA= 0.1. Regression indexes ranged from 0.6 (ISB1ßDEs) to 0.8 (ISB8ßDEs), being thus higher than 0.4, which indicates that these items are constitutive components of their scales.

The diagnosis of burnout syndrome according to already established criteria,^7^ could be inferred in 628 physicians, accounting for 59.4% of the 1,058 who completed the instrument; 266 men (42.4%) and 362 women (57.6%).

The results obtained with the UWES revealed that most professionals had very high, high, or medium levels of engagement. Approximately 40% of them showed very high levels in the four dimensions assessed. Means ranged from 3.64 to 4.11, with the following distribution across dimensions: VI (M = 3.6; SD = 1.3), DE (M = 3.9; SD = 1.1), AB (M = 4.1; SD = 1.0), and overall engagement (M = 3.9; DP=l.l) indicating mostly moderate and high levels of work engagement. Results are described in Table 3.

**Table 3:** Mean, SD, Cronbach’s alpha, and frequency distribution of UWES scores

Scale	Mean	SD	α	Total (n)	Very high (%)	High (%)	Average (%)	Low (%)	Very low (%)
VI	3.6	1.3	0.9	1,026	38.2	11.9	33.4	8.1	8.4
DE	3.9	1.1	0.9	1,021	38.8	19.3	32.9	7.4	1.6
AB	4.1	1.0	0.75	917	45.0	27.2	23.6	2.2	2.1
ENG	3.9	1.1	0.9	977	42.3	20.0	28.2	7.6	1.9

AB = Absorption; DE = Dedication; ENG = overall work engagement; SD = standard deviation; UWES = Utrecht Work Engagement Scale; VI = Vigor; α = Cronbach’s alpha.

Reliability rates were adequate, ranging from 0.8 (AB) to 0.9 (UWES). Results of confirmatory factor analyses showed to be adequate, attesting the quality of the model for the present sample: CFI = 0.9; AGFI=0.9; RMSEA = 1.7. Regression indexes ranged from 0.5 (UWES9ßAB) to 0.9 (UWES2ßVI), being thus higher than 0.4, which indicates that these items are constitutive components of their scales. With regard to mean UWES scores, they were higher than those presented in the data from instrument’s manual.^16^

The Pearson correlation test between the scales showed that all results were statistically significant and coherent with the theoretical premise of a positive correlation between stress and burnout and a negative correlation between work engagement and these variables.^19,22^

Pearson correlation of ISE scores was 0.6. With regard to the ISB, the highest value was found between COP and CON (*r* = −0.7), and the lowest between NOC and PA (*r* = −0.4). With regard to the UWES, all correlations were higher than *r* = 0.8 (VI-AB).

With regard to inter-instrument correlations, the highest value was found between PA and DE (*r* = 0.8), which indicates that PA tends to be closely related to dedication to work activities. Conversely, the lowest correlation was observed between NOC and AB at work (*r* = −0.3), indicating that the strength of the correlation between these variables, although significant, is the weakest among those analyzed.

## DISCUSSION

One of the limitations of the study was the number of questionnaires returned, which was below the expected, a fact that may be explained by the size of the questionnaires, since each inventory consisted of several questions requiring attention from respondents; in general, physicians do not usually volunteer their limited time for that.

Another limitation was the non-probabilistic sampling technique used in the study, which prevents generalization of the results for the population of physicians in Paraná.

It is worth noting the high number of professionals who were or had already been on psychiatric or psychotherapeutic treatment and of those using continuous and/or controlled medications, suggesting that most physicians in the sample are experiencing physical and/or emotional stress. This fact may have influenced their decision of answering the questionnaire completely; for them, the study may have represented a cry for help. Those who did not answer the questionnaire were not interested in the subject or did not want to expose themselves, despite being informed about the confidential nature of the research.

Another important aspect is that most physicians (74.5%) believed that medical practice was a source of stress and that almost a half (46.8%) had already thought in changing profession. Unfortunately, it was not possible to correlate these findings with demographic data, because working bonds, hours, and places varied significantly, thus hampering the compilation of answers and the comparison of test results.

The analyses of answers for the ISE allowed to conclude that participants are in a stressful situation, with a very high percentage of them showing high levels of PsyS and/or PhyS, with higher mean scores compared to those of studies with other professional categories.^19,22^

It is known that the strategies used by individuals to adapt to a stressful situation, either cognitive or material, are not always successful.^3^ When they fail, it may result in diseases such as burnout syndrome and depression, among others.^1,2^ The results obtained by the ISB may be an indicative of this process. The fact that most physicians was relatively young, with a mean age of 37 years old, thus at the beginning of their career, partially explains these findings, since occupational stress is more frequent in this phase.^1,2,5^ However, these findings do not rule out the possibility that older professionals and those with longer professional experience develop signs and symptoms of occupational diseases such as those investigated in the present study. Only 8.7% of participants reported being 60 years or older, and it was not possible to establish a relationship between belonging to this age group and having the aforementioned diseases or other chronic diseases.

Most participants reported being in a stable relationship at the time of the research, an aspect that would have a protective effect against stress, as well as regular physical activity, which, however, was reported by only 20% of respondents.

The results for the tests of the first part of the ISB indicated that an unfavorable work environment predominated in the sample, as shown by high scores in NOC, which may undesirably interfere with work activities and contribute to the development of stress and burnout.

Burnout indicators had higher mean scores than those found in the study by Benevides-Pereira et al.^23^ with 701 professionals from industries. The diagnosis of burnout syndrome could be inferred in almost 60% of physicians and was more frequent in women (362; 57.6% of 628). This high percentage of burnout suggests that the work environment is compromising the health of these professionals, as shown by the predominance of NOC, considered factors preceding the syndrome.^23^ It is necessary to develop occupational health strategies to ensure safety and maintenance of health, technical quality, and satisfactory productivity for these professionals.

In a study conducted by Lima et al.^24^ including 134 (22.3%) of the 600 physicians from a hospital in Rio de Janeiro, Brazil, the percentage of burnout was 10%, a value considerably lower than that reported in the present study, although 82.1% of physicians in their study showed high levels in at least one of the dimensions of the syndrome. Schwartz^25^ published the results of a research with 1,838 Brazilian physicians, in which 8% of professionals reported suffering from depression and 26% from burnout, whereas 11% reported suffering from both conditions; this study was based on self-reported diagnosis.

The results for the UWES^16^ indicated that, in general, professionals were engaged to work. Approximately 40% of them had very high levels in the four dimensions assessed: VI, DE, AB, and overall engagement, with AB and overall engagement showing the highest means. Similar results were obtained by Teixeira et al.^26^ in a study conducted in Brazil using the same instrument and including 36 pediatrics residents, with higher means for the DE dimension. International studies with the

UWES, such as an investigation with 123 surgeons in Germany, revealed that participants showed higher levels of overall engagement than those reported in the present study, with M = 4.4; SD = 0.9,^27^ results more desirable related to the construct. In a sample of 111 workers from a high complexity surgical unit in Cali, Colombia, Ortiz & Jaramillo^28^ found higher mean scores for overall engagement (M = 4.9), scores also higher than those reported for the sample from the state of Paraná, Brazil. These authors^28^ also point out that these findings indicate a positive mental state, with commitment, persistence, and work enthusiasm, but they remember that it does not imply ruling out the issue of occupational stress.

The findings obtained in the present research coincide with those of other authors who also questioned why, despite precarious working conditions and high levels of stress, most physicians are always willing to work, sometimes working overtime, a fact that should be further analyzed.^14,24,29^

Engagement may have contributed to protecting against burnout and in strategies to cope with stress, a desirable aspect in occupational health psychology, indicating a direction and the concrete possibility of transforming work processes to become them health promoter.^1^ Engagement reinforces the effects of the available organizational resources, contributing to increase in performance and organization well-being.^17,18^

There was a positive correlation between stress and burnout, and a negative correlation of work engagement with stress and burnout; a result already expected according to the theoretical premise.^19,22^ Inter-instrument correlations showed that PA is associated with dedication to work activities and that NOC are negative correlated with AB at work.

It is important that professional organizations and health managers reflect on and suggest health promotion and well-being actions of these professionals. In general, the few intervention programs that may improve physicians’ health state act only at the individual levels, advising on how to deal with and reduce stress.^15^ Organizational intervention programs are rare and should be encouraged.

Ensuring occupational safety should be the goal of professional organizations and health managers, paying attention to factors such as number of working hours, high demand of activities, sleep deprivation, and degree of autonomy, which affect occupational health and put professionals under pressure, leading them to EE, which has a confirmed association with depression.^14^ There is the need for studies on the process of medical work in its multiple forms, when searching for an improvement in quality of life and health of these professionals, which will certainly have a positive influence on their practices.^1,15^

Further studies are recommended in order to better explain these results. It may be interesting to apply the tests in a random sample and, as much as possible, applying each instrument in different groups, in order to increase physicians’ adherence, since long questionnaires are associated with withdrawal.

It is suggested to analyze the associations between test results and demographic characteristics, in to investigate a possible cause-effect relationship, an aspect that was hampered in this study.

## CONCLUSIONS

1. Although the number of physicians who answered the tests were below the expected, the aim of evaluating stress, burnout, and work engagement was achieved;
2. Most physicians in the sample experienced signs of stress;
3. NOC prevailed in the work environment;
4. There was a high number of individuals with burnout in the sample population;
5. Work engagement was frequent, despite the predominance of stress and NOC;
6. The instruments showed adequate reliability indexes and confirmatory factor analyses, as well significant correlations between the scales, denoting that the constructs were associated for the present sample.

## HOMAGE

The author Ana Maria Benevides Pereira participated actively in the development of the article, but unfortunately passed away before its publication. We thank her for her valuable contributions to this study.

## References

[1] Dias EC (2015). Condições de trabalho e saúde dos médicos: uma
questão negligenciada e um desafio para a Associação Nacional de
Medicina do Trabalho. Rev Bras Med Trab.

[2] Nascimento Sobrinho CL, Carvalho FM, Bonfim TAS, Cirino CAS, Ferreira ISF (2006). Condições de trabalho e saúde mental dos
médicos de Salvador, Bahia, Brasil. Cad Saude Publica.

[3] Sampaio ARC (2011). Burnout, estratégias de coping e qualidade de vida nos profissionais de
saúde.

[4] Sparrenberger F, Dos Santos I, Lima RC (2003). Epidemiologia do distress psicológico: estudo transversal de base
populacional. Rev Saude Publica.

[5] World Health Organization (WHO) (2020). Occupational health: stress at the workplace [Internet].

[6] Benevides-Pereira AMT, Gil-Monte P, Moreno-Jiménez B (2007). El síndrome de quemarse por el trabajo (burnout) en grupos ocupacionales de
riesgo.

[7] Benevides-Pereira AMT (2015). Elaboração e validação do ISB –
Inventário de avaliação da Síndrome de
Burnout. Bol Psicol.

[8] Benevides-Pereira AMT, Levy GCTM, Nunes Sobrinho FP (2010). A Síndrome de Burnout em professores do ensino regular: pesquisa,
reflexões e enfrentamento.

[9] Maslach C, Leiter MP (2016). Understanding the burnout experience: recent research and its implications
for psychiatry. World Psychiatry.

[10] Paolini HO, Bertram B, Hamilton T (2013). Antidotes to Burnout: fostering physician resiliency, well-being, and
holistic development. MBA.

[11] Bianchi R, Schonfeld IS, Laurent E (2015). Burnout-depression overlap: a review. Clin Psychol Rev.

[12] Schonfeld IS, Bianchi R (2016). Burnout and depression: two entities or one?. J Clin Psychol.

[13] Oquendo MA, Bernstein CA, Mayer LES (2019). A key differential diagnosis for physicians-major depression or
burnout?. JAMA Psychiatry.

[14] Tyssen R (2007). Health problems and the use of health services among Physicians: a review
article with particular emphasis on Norwegian studies. Ind Health.

[15] Shanafelt TD (2009). Enhancing meaning in work: a prescription for preventing physician burnout
and promoting patient-centered care. JAMA.

[16] Schaufeli WB, Bakker A (2003). Utrecht Work Engagement Scale (UWES).

[17] Porto-Martins PC, Basso-Machado PG, Benevides-Pereira AMT (2013). Engajamento no trabalho: uma discussão
teórica. Fractal Rev Psicol.

[18] Schaufeli WB (2012). Work engagement: what do we know and where do we go?. Rom J Appl Psychol.

[19] Porto-Martins PC (2016). Salud Laboral en Teleoperadores: un enfoque en el estrés, el
síndrome de burnout, la resiliencia y el engajamento en el trabajo.

[20] Benevides-Pereira AMT, Moreno-Jiménez B (2000). O burnout em psicólogos [Report] Universidade Estadual de
Maringá.

[21] Agnst R, Benevides-Pereira AMT, Porto-Martins PC (2009). UWES Utrecht Work Engagement Scale.

[22] Machado PGB (2014). Indicadores de Salud Enfermidad Laboral en una Muestra Multiocupacional.

[23] Benevides-Pereira AMT, Machado PGB, Porto-Martins PC, Carrobles JA, Siqueira JO (2017). Confirmatory factor analysis of the ISB – Burnout Syndrome
inventory. Psychol Commun Health.

[24] Lima CRC, Sepúlveda JLM, Lopes PHTNP, Fajardo HSR, De Sousa MM, Ferreira Júnior MC (2018). Prevalência da síndrome de burnout em médicos
militares de um hospital público no Rio de Janeiro, Brasil. Rev Bras Med Trab.

[25] Schwartz L (2019). Estilo de vida e burnout médico no Brasil.

[26] Teixeira PR, Lourenção LG, Gazzeta A, Gonzalez EG, Rotta DS, Pinto MH (2017). Engajamento no trabalho em residentes médicos de
Pediatria. Rev Bras Educ Med.

[27] Mache S, Vitzthum K, Klapp B, Danzer G (2014). Surgeons’ work engagement: influencing factors and relations to job and
life satisfaction. Surgeon.

[28] Ortiz FA, Jaramillo VA (2012). Factores de riesgo psicosocial y compromiso (engajamento) con el trabajo en
una organización del sector salud de la ciudad de Cali, Colombia. Acta Colomb Psicol.

[29] Moreira HA, Souza KN, Yamaguchi MU (2018). Síndrome de Burnout em médicos: uma revisão
sistemática. Rev Bras Saude Ocup.

